# Modelling temporal data in knowledge graphs: a systematic review protocol

**DOI:** 10.12688/hrbopenres.13403.1

**Published:** 2021-09-10

**Authors:** Sepideh Hooshafza, Fabrizio Orlandi, Rachel Flynn, Louise McQuaid, Gaye Stephens, Laura O'Connor

**Affiliations:** 1 Health Information and Quality Authority (HIQA), Cork, Ireland; 2 School of Computer Science and Statistics, Trinity College Dublin, Dublin, Ireland

**Keywords:** Knowledge graph, temporal data, resource description framework

## Abstract

**Background:** The benefits of having high-quality healthcare data are well established. However, high-dimensionality and irregularity of healthcare data pose challenges in their management. Knowledge graphs have gained increasing popularity in many domains, as a method for representing data to overcome such challenges. However, little is known about their suitability for use with healthcare data.

One important factor in representing data is “time”. Data with time related attributes are considered temporal data. Temporal data are frequently observed in healthcare and the management of rapidly changing patient data is an ongoing challenge. Traditionally, data models have focused on presenting static data and do not account for temporal data. Temporal data models ensure time consistency in data models and assist analysing the history of data and predicting the future trends in data. Knowledge graphs can include temporal data models and are therefore of interest to the field of healthcare data management.

As such, the herein aim is to outline a protocol for an inter-disciplinary systematic review of approaches, applications and challenges in modelling temporal data in knowledge graphs so that we can inform the application of knowledge graphs to healthcare data.

**Method:** The research questions is, what are the existing approaches in modelling temporal data in knowledge graphs. Two sub-questions on applications, and challenges will also be evaluated. ACM digital library, IEEEXplore and ScienceDirect will be searched for this review. The search will be limited to peer-reviewed literature referring to knowledge graphs based on Resource Description Framework (RDF). A narrative synthesis of the papers will be conducted.

**Conclusion:** The findings of this systematic review will be useful for data engineers to better represent data and perform analytics through temporal data modelling. They can be applied in the context of healthcare data and the current challenges faced in managing rapidly changing patient data.

## Introduction

The benefits of having high-quality, up-to-date, usable healthcare data are well established ^1^. The healthcare data comes from different sources such as hospitals, patient registries, clinics and are collected over time ^2^. These sources generate large amounts of healthcare data such as patients’ medical histories, physicians’ notes, prescriptions, laboratory results and scan reports ^3^. High-dimensionality, irregularity and sparsity of healthcare data pose challenges in their management, processing and usability ^4^. Moreover, the volume of data generated in healthcare setting are increasing rapidly and makes it complicated for managing and analysing data ^2^. As such, there is a need for effective methods for healthcare data storage and representation.

In recent years, knowledge graphs (KGs) have been used in academic and industry as a method for managing and representing data ^5, 6^. They have attracted attention in several application areas including natural language processing, question answering machines, recommendation system ^7^. However, little is known about the use or suitability for use with healthcare data.

KGs are defined as a semantic network comprising entities (nodes) and relationships (edges) ^8^. There are two main types of KGs adhering to the Resource Description Framework (RDF) data model or the property graphs model ^9^. RDF is a standard language for data representation and interchange on the Web ^10^. RDF graphs are popular in practice and follow the World Wide Web Consortium (W3C) standards ^11^.

An important factor in storing data is “time” ^12^. Time-varying data (also called temporal data) are data that have a time related attribute. Temporal data are created by including timestamps for the data values ^13, 14^. Timestamps in a data model are mostly used to indicate time points (valid time) in which the data values are valid and transaction time in which the data values are recorded ^13, 15, 16^. Previous studies in the field of knowledge graphs focused on static data, however, methods to deal with and capture the variation and development of data over time, is of high importance ^17, 18^. Storing data by considering time varying knowledge, ensures time consistency in a data model, improve performance of KG models, and assist analysing the history of data and predicting the future trends in data as well ^12, 15^.

The herein aim is to outline a protocol for a systematic review to explore existing approaches, applications and challenges in modelling temporal data in knowledge graphs. The results of the systematic review will inform data engineers when modelling temporal data. This study is particularly pertinent in the context of health data and the current challenges faced in managing rapidly changing patient data for example, for the development of centralised patient records and patient portals ^19^.

## Protocol

### Research methodology

This systematic review is based on the guidelines and procedures for systematic reviews within the software engineering domain ^20, 21^.

The procedure that will be undertaken in this study is as follows:

1. Formulating the research questions;
2. Selecting information sources (digital libraries) on which to perform search;
3. Defining search concepts and keywords;
4. Application of search terms on databases;
5. Considering inclusion and exclusion criteria for selection of studies;
6. Quality appraisal of the included studies;
7. Synthesis of data.

### Research question

Major research question: What are the existing approaches in modelling temporal data in knowledge graphs?

Sub-questions:

1. What are the existing applications of temporal data models in knowledge graphs?
2. What are the existing challenges with modelling temporal data in knowledge graphs?

### Information sources

Searches will be carried out on the following databases: ACM Digital Library ^22^, IEEE Xplore Digital Library ^23^, and ScienceDirect ^24^. The bibliographies of the included full-text articles will be searched for relevant articles. Searching of forward citations will also be conducted to identify other potential material for inclusion.

### Search strategy

The search terms to be used, are set out in Table 1.

**Table 1.** Search terms for a systematic review on modelling temporal data in knowledge graphs.

Concepts	Search terms
Knowledge graph	“Knowledge graph” OR RDF OR “resource description framework”
Temporal	Temporal OR dynamic OR evolution OR time
Applied model	*present OR annotate OR model OR schema OR standard OR framework OR structure OR application OR applied

The search query to be used for ACM digital library, as an example of the search strategy, is:

“(“Knowledge graph” OR rdf OR “resource description framework”) AND (Temporal OR dynamic OR evolution OR time) AND (*present OR annotate OR model OR schema OR standard OR framework OR structure OR application OR applied)"

### Criteria for inclusion

No limits will be applied to articles for inclusion in terms of publication date or language.

Articles will be included if they:

•  Refer to approaches in modelling temporal data in KGs

    OR

•  Discuss applications of temporal data modelling in KGs

    OR

•  Address challenges of temporal data modelling in KGs

    AND

•  Refer to knowledge graphs based on Resource Description Framework (RDF)

Studies will be excluded if they refer to knowledge graphs based on frameworks other than Resource Description Framework (RDF).

### Types of study to be included

Peer-reviewed literature will be selected to be reviewed in this study. Given the nature of the topic under review, it is anticipated that the studies will mostly fall into the category of original research articles.

### Software

Covidence by Veritas Health Innovation Ltd, a web-based software platform for systematic review management, will be used for screening articles ^25^. EndNote X8.2 by PDF Tron™ Systems Inc. will be used to manage the bibliography ^26^. Microsoft Excel will be used to manage the extracted data.

### Screening

All retrieved articles from the selected information sources will be imported into Covidence. Duplicate references will be removed. Two reviewers will independently screen the titles and abstracts against the inclusion/exclusion criteria. Any disagreements on inclusion/exclusion will be firstly resolved by discussion. Any disagreements not resolved by discussion will be resolved by a third author. Forward citation and hand-searching of bibliographies of included studies will be performed and any relevant studies identified will be included. The Preferred Reporting Items for Systematic reviews and Meta-Analyses (PRISMA) statement will be used to report the search and study selection process ^27^.

### Quality appraisal

A quality appraisal tool will be used to inform weighting of discussion based on the quality of the included articles. The quality appraisal checklist proposed in the Guidelines for performing systematic literature reviews in software engineering will be used for this purpose ^20^. Two reviewers will independently appraise the quality of selected articles. If agreement cannot be reached, a third researcher will assess the studies to come to a consensus. Articles will not be excluded based on their quality.

### Data extraction

A data extraction table will be developed in Covidence to structure and categorise the findings (See extended data). The data to be extracted in the table includes study ID, study title, author(s), publication type, year of publication, journal/conference title, setting, modelling approaches, applications, and challenges in modelling temporal data in knowledge graphs. Once the table is completed for all the final included articles, the table will be exported to Excel and data synthesis will be conducted.

The data extraction table will be piloted on three articles by two researchers to ensure appropriateness of the included data extraction fields against the data provided in articles and mutual understanding of the fields. The data extraction table will be updated at this point, if required.

### Data synthesis

The information will be manually extracted from each included article. Articles will be read in full by one researcher and the data extracted directly into the data extraction table. A second researcher will independently complete data extraction for 10% of the identified articles for quality assurance purposes. A narrative synthesis will be performed to analyse the articles. To facilitate the visualization of the information, the synthesis of the extracted data will be presented in different forms including tables, graphs and other artefacts.

### Dissemination of information

The systematic review will be submitted to an academic journal on completion. Conference abstracts arising out of the systematic review will also be submitted to appropriate conferences for presentation.

### Strengths and limitations

To the best of the authors’ knowledge, this review will be the first to systematically describe temporal data modelling in knowledge graphs. In addition, the methodological approach allows for a comprehensive exploration of modelling approaches, applications, and challenges of temporal data modelling in knowledge graphs. A further strength of this review is that the search is not limited to the field of healthcare information. It has been designed so that we can gain learning from across the disciplines and use that to inform practice in health information management.

In terms of limitations, due to the multiplicity of concepts and keywords used in the literature, there is a risk that some relevant studies may not be retrieved. This risk has been reduced by evaluating a range of studies in preliminary searches to ensure that equivalent words and phrases are included in the search terms. Furthermore, the inclusion of hand-searching of bibliographies and forward citation searching is designed to, in part, overcome this limitation.

## Conclusions

The purpose of conducting this review is to identify existing approaches and applications of modelling temporal data in knowledge graphs and to identify challenges of modelling temporal data in knowledge graphs. The findings of the systematic review will be of interest to organisations working in the field of data science. They can also inform quality improvement initiatives for health information system service providers and help generate new ideas in temporal healthcare data modelling and develop data analytics solution based on temporal healthcare data. This will be beneficial in addressing the current challenges faced in managing rapidly changing patient data.

## Study status

Searching the information sources using the search terms outlined in Table 1 has commenced.

## Data availability

### Underlying data

No data are associated with this article.

### Extended data

Figshare: Data Extraction Table_Systematic Review_SH 2021.docx. https://doi.org/10.6084/m9.figshare.16528308 ^28^

This protocol contains the following extended data: Data Extraction Table_Systematic Review_SH 2021.docx. (Data extraction table)

### Reporting guidelines

Figshare: PRISMA-P checklist for “Modelling temporal data in knowledge graphs: a systematic review protocol”. https://doi.org/10.6084/m9.figshare.16499031 ^29^

Data are available under the terms of the Creative Commons Attribution 4.0 International license (CC-BY 4.0).
