# Modelling temporal data in knowledge graphs: a systematic review protocol

**DOI:** 10.12688/hrbopenres.13403.2

**Published:** 2022-08-02

**Authors:** Sepideh Hooshafza, Fabrizio Orlandi, Rachel Flynn, Louise McQuaid, Gaye Stephens, Laura O'Connor

**Affiliations:** 1 Health Information and Quality Authority (HIQA), Cork, Ireland; 2 The SFI ADAPT Research Centre for AI-Driven Digital Content Technology, School of Computer Science and Statistics, Trinity College Dublin, Dublin, Ireland

**Keywords:** Knowledge graph, temporal data, resource description framework

## Abstract

**Background:** The benefits of having high-quality healthcare data are well established. However, high-dimensionality and irregularity of healthcare data pose challenges in their management. Knowledge graphs have gained increasing popularity in many domains, as a method for representing data to overcome such challenges. However, little is known about their suitability for use with healthcare data.

One important factor in representing data is “time”. Data with time related attributes are considered, temporal data. Temporal data are frequently observed in healthcare and the management of rapidly changing patient data is an ongoing challenge. Traditionally, data models have focused on presenting static data and do not account for temporal data. Temporal data models ensure time consistency in data models and assist analysing the history of data and predicting the future trends in data. Knowledge graphs can include temporal data models and are therefore of interest to the field of healthcare data management.

As such, the herein aim is to outline a protocol for an inter-disciplinary systematic review of approaches, applications and challenges in modelling temporal data in knowledge graphs so that we can inform the application of knowledge graphs to healthcare data.

**Method:** The research questions is, what are the existing approaches in modelling temporal data in RDF based knowledge graphs. Two sub-questions on applications, and challenges will also be evaluated. ACM digital library, IEEE Xplore and Scopus will be searched for this review. The search will be limited to peer-reviewed literature referring to knowledge graphs based on Resource Description Framework (RDF). A narrative synthesis of the papers will be conducted.

**Conclusion:** The findings of this systematic review will be useful for data engineers to better represent data and perform analytics through temporal data modelling. They can be applied in the context of healthcare data and the current challenges faced in managing rapidly changing patient data.

## Introduction

The benefits of having high-quality, up-to-date, usable healthcare data are well established ^1^. The healthcare data come from different sources such as hospitals, patient registries, clinics and are collected over time ^2^. These sources generate large amounts of healthcare data such as patients’ medical histories, physicians’ notes, prescriptions, laboratory results and scan reports ^3^. High-dimensionality, irregularity and sparsity of healthcare data pose challenges in their management, processing and usability ^4^. Moreover, the volume of data generated in healthcare setting are increasing rapidly and makes it complicated for managing and analysing data ^2^. As such, there is a need for effective methods for healthcare data storage and representation.

An important factor in storing data is “time” ^5^. Time-varying data (also called temporal data) are data that have a time related attribute. Temporal data are created by including timestamps for the data values ^6, 7^. Timestamps in a data model are mostly used to indicate time points (valid time) in which the data values are valid and transaction time in which the data values are recorded ^6, 8, 9^

Most healthcare data are temporal in nature ^10^. Most of the clinical data such as patients’ symptoms, laboratory test results, data from health monitoring devices, and various clinical measurements, are associated with a time in which the data is valid (valid time). Furthermore, capturing the time that the data are inserted into the database (transaction time) is required for legal purposes, treatment purposes or for justifying physicians’ decisions ^10^. As a simple example, suppose a patient has been hospitalised on February 18th at 8:00 am. This is the valid time for the hospitalisation time. This piece of information is inserted into hospital database on Feb 18 at 12:00 pm. This time is called the transaction time ^10^. Capturing both transaction time and valid time is important to ensure that the decision making process is accurate and valuable and is beneficial in designing decision support systems ^11^.

Improved management of temporal healthcare data would benefit healthcare practitioners in information retrieval, healthcare decision making and support patient care ^12^. Temporal data can assist in exploring temporal patterns in diseases and identifying disease progression. It would help in assessing patients’ clinical history, finding possible causes of clinical events over time, and predicting future trends and events based on past and current clinical data ^2^. Hence, the representation and query of temporal data has become a priority research area and efficient solutions are needed to model and store temporal data in healthcare settings ^13, 14^. 

Most of the solutions implemented so far for storing, and representing valid time and transaction time are based on relational databases. The developers of clinical database systems have some difficulty managing time values in relational databases for highly connected data. Firstly, modelling relationships and semantics between data items is not easy to implement in relational databases ^15^. Secondly, for the large volume of data coming from heterogeneous sources, it is difficult to query a large number of joining tables ^16^. As such, knowledge graphs (KGs) are potential solution to these problems.

In recent years, knowledge graphs (KGs) have been used in academic and industry as a method for managing and representing data ^17, 18^. They have attracted attention in several application areas including natural language processing, question answering machines, recommendation system ^19^.

KGs are defined as a semantic network comprising entities (nodes) and relationships (edges) ^14^. There are two main types of KGs adhering to the Resource Description Framework (RDF) data model or the property graphs model ^20^. For a number of reasons, the focus of this review is on RDF based knowledge graphs. RDF is a standard language for data representation and interchange on the Web ^21^. RDF graphs are popular in practice and follow the World Wide Web Consortium (W3C) standards ^22^. A community of practice and supporting tools have developed around the RDF and related semantic web standards. Many standards based public data models (called ontologies) are available to support and guide RDF KG enhancement and development.

Previous studies in the field of knowledge graphs focused on static data, however, methods to deal with and capture the variation and development of data over time, is of high importance and little is known about presenting temporal data in knowledge graphs ^23, 24^. Storing data by considering time varying knowledge, ensures time consistency in a data model, improve performance of KG models, and assist analysing the history of data and predicting the future trends in data as well ^5, 8^.

The herein aim is to outline a protocol for a systematic review to explore existing approaches, applications and challenges in modelling temporal data in knowledge graphs. The results of the systematic review will inform data engineers and others of the feasibility and challenges involved when modelling temporal data. In healthcare, the findings of this study will assist in modelling patient data, data from health monitoring devices and data collected within services over time. There are different international health information modelling standards including OpenEHR, HL7 which are used for modelling information at high level and for semantic interoperability purposes. In this review our focus is on the modelling in the data level, where we can model data to store them in the database and query them ^25, 26^. International health information modelling standards can be used to get a high level overview of the concept with its included information items and can be used for designing different concepts in knowledge graphs but they are not replaceable.

The management of rapidly changing patient data and types of data sets is an ongoing challenge. This study focuses on the approaches and challenges in KG modelling to support this management ^2, 12^. 

## Protocol

### Research methodology

This systematic review is based on the guidelines and procedures for systematic reviews within the software engineering domain ^27, 28^.

The procedure that will be undertaken in this study is as follows:

1. Formulating the research questions;
2. Selecting information sources (digital libraries) on which to perform search;
3. Defining search concepts and keywords;
4. Application of search terms on databases;
5. Considering inclusion and exclusion criteria for selection of studies;
6. Quality appraisal of the included studies;
7. Synthesis of data.

### Research question

Major research question: What are the existing approaches in modelling temporal data in RDF based knowledge graphs?

Sub-questions:

1. What are the existing applications of temporal data models in RDF based knowledge graphs?

2.What are the existing challenges with modelling temporal data in RDF based knowledge graphs?

### Information sources

Searches will be carried out on the following databases: ACM Digital Library ^29^, IEEE Xplore Digital Library ^30^, and Scopus ^31^. The bibliographies of the included full-text articles will be searched for relevant articles. Searching of forward citations will also be conducted to identify other potential material for inclusion.

### Search strategy

The search strategy was developed using the PEO (Population (Context), Exposure, and Outcome) framework as follows ^32^, Population (Context): knowledge graphs, Exposure: Time, and Outcome: Applied model. The search concepts and keywords based on PEO framework are set out in Table 1.

**Table 1.** Search terms for a systematic review on modelling temporal data in knowledge graphs.

PEO framework	Concepts	Search terms
Population (context)	Knowledge graph	“Knowledge graph” OR RDF OR “resource description framework”
Exposure	Temporal	Temporal OR dynamic OR evolution OR time
Outcome	Applied model	*present OR annotate OR model OR schema OR standard OR framework OR structure OR application OR applied

The search query to be used for ACM digital library and Scopus, is:

“((“Knowledge graph” OR rdf OR “resource description framework”) AND (Temporal OR dynamic OR evolution OR time) AND (*present OR annotate OR model OR schema OR standard OR framework OR structure OR application OR applied))"

Since IEEE Xplore Digital Library does not accept using parenthesis in the advanced search, the search query to be used is:

*“Knowledge graph” OR rdf OR “resource description framework”*

*AND*

*Temporal OR dynamic OR evolution OR time*

*AND*

*present OR annotate OR model OR schema OR standard OR framework OR structure OR application OR applied

### Criteria for inclusion

No limits will be applied to articles for inclusion in terms of publication date or language.

Articles will be included if they:

•  Refer to approaches in modelling temporal data in KGs

    OR

•  Discuss applications of temporal data modelling in KGs

    OR

•  Address challenges of temporal data modelling in KGs

    AND

•  Refer to knowledge graphs based on Resource Description Framework (RDF)

Studies will be excluded if they refer to knowledge graphs based on frameworks other than Resource Description Framework (RDF).

### Types of study to be included

Peer-reviewed literature will be selected to be reviewed in this study. Given the nature of the topic under review, it is anticipated that the studies will mostly fall into the category of original research articles.

### Software

Covidence by Veritas Health Innovation Ltd, a web-based software platform for systematic review management, will be used for screening articles ^33^. EndNote X8.2 by PDF Tron™ Systems Inc. will be used to manage the bibliography ^34^. Microsoft Excel will be used to manage the extracted data.

### Screening

All retrieved articles from the selected information sources will be imported into Covidence. Duplicate references will be removed. Two reviewers will independently screen the titles and abstracts against the inclusion/exclusion criteria. Any disagreements on inclusion/exclusion will be firstly resolved by discussion. Any disagreements not resolved by discussion will be resolved by a third author. Forward citation and hand-searching of bibliographies of included studies will be performed and any relevant studies identified will be included. The Preferred Reporting Items for Systematic reviews and Meta-Analyses (PRISMA) statement will be used to report the search and study selection process ^35^.

### Quality appraisal

A quality appraisal tool will be used to inform weighting of discussion based on the quality of the included articles. The quality appraisal checklist proposed in the Guidelines for performing systematic literature reviews in software engineering will be used for this purpose ^27^. Two reviewers will independently appraise the quality of selected articles. If agreement cannot be reached, a third researcher will assess the studies to come to a consensus. Articles will not be excluded based on their quality.

### Data extraction

A data extraction table will be developed in Covidence to structure and categorise the findings (See extended data). The data to be extracted in the table includes study ID, study title, author(s), publication type, year of publication, journal/conference title, setting, modelling approaches, applications, and challenges in modelling temporal data in knowledge graphs. Once the table is completed for all the final included articles, the table will be exported to Excel and data synthesis will be conducted.

The data extraction table will be piloted on three articles by two researchers to ensure appropriateness of the included data extraction fields against the data provided in articles and mutual understanding of the fields. The data extraction table will be updated at this point, if required.

### Data synthesis

The information will be manually extracted from each included article. Articles will be read in full by one researcher and the data extracted directly into the data extraction table. A second researcher will independently complete data extraction for 10% of the identified articles for quality assurance purposes. A narrative synthesis will be performed to analyse the articles. To facilitate the visualization of the information, the synthesis of the extracted data will be presented in different forms including tables, graphs and other artefacts.

### Dissemination of information

The systematic review will be submitted to an academic journal on completion. Conference abstracts arising out of the systematic review will also be submitted to appropriate conferences for presentation.

### Strengths and limitations

To the best of the authors’ knowledge, this review will be the first to systematically describe temporal data modelling in knowledge graphs. In addition, the methodological approach allows for a comprehensive exploration of modelling approaches, applications, and challenges of temporal data modelling in knowledge graphs. A further strength of this review is that the search is not limited to the field of healthcare information. It has been designed so that we can gain learning from across the disciplines and use that to inform practice in health information management.

In terms of limitations, due to the multiplicity of concepts and keywords used in the literature, there is a risk that some relevant studies may not be retrieved. This risk has been reduced by evaluating a range of studies in preliminary searches to ensure that equivalent words and phrases are included in the search terms. Furthermore, the inclusion of hand-searching of bibliographies and forward citation searching is designed to, in part, overcome this limitation.

## Conclusions

The purpose of conducting this review is to identify existing approaches and applications of modelling temporal data in knowledge graphs and to identify challenges of modelling temporal data in knowledge graphs. The findings of the systematic review will be of interest to organisations working in the fields of data science, data engineering, informatics and other similar areas. They can also inform quality improvement initiatives for health information system service providers and help generate new ideas in temporal healthcare data modelling and develop data analytics solution based on temporal healthcare data. This will be beneficial in addressing the current challenges faced in managing rapidly changing patient data.

## Study status

Searching the information sources using the search terms outlined in Table 1 has commenced.

## Data availability

### Underlying data

No data are associated with this article.

### Extended data

Figshare: Data Extraction Table_Systematic Review_SH 2021.docx. https://doi.org/10.6084/m9.figshare.16528308 ^36^

This protocol contains the following extended data: Data Extraction Table_Systematic Review_SH 2021.docx. (Data extraction table)

### Reporting guidelines

Figshare: PRISMA-P checklist for “Modelling temporal data in knowledge graphs: a systematic review protocol”. https://doi.org/10.6084/m9.figshare.16499031 ^37^

Data are available under the terms of the Creative Commons Attribution 4.0 International license (CC-BY 4.0).

## Supplementary material

This study does not contain any supplementary material.
