# Supplementary material for: Modelling temporal data in knowledge graphs: a systematic review protocol
Source: HRB Open Res. 2022 Aug 2;4:101. Originally published 2021 Sep 10. [Version 2] doi: 10.12688/hrbopenres.13403.2 (PMC10907873; doi:10.12688/hrbopenres.13403.2)
Supplement: Peer review history

# Reviewer report: Reviewer response for version 2

**DOI:** 10.21956/hrbopenres.14859.r38059

**Published:** 2024-03-01

**Authors:** Enrique Iglesias

**Affiliations:** 1 Leibniz University of Hannover, Hannover, Germany

**General Comments**

**Summary**

 This paper proposes a study that analyses existing approaches, applications, and challenges in modeling temporal data. The purpose of this study is to determine which existing approaches and works that will help inform engineers on the challenges of modeling temporal data. Additionally, this work seeks to inform healthcare workers about the benefits of using temporal data in knowledge graphs.

 This paper describes the study the authors conducted to find existing works illustrating the benefits and challenges of modeling temporal data. The authors used multiple databases like ACM Digital Library, IEEE Xplore Digital Library, and Scopus to extract relevant works. Additionally, the authors search the references of the relevant papers for additional papers that will enrich the study.

 This paper has two main negative points. First, some sentences in the introduction need to be rewritten. Some sentences could be more understandable. For example, “A community of practice and supporting tools have developed around the RDF and related semantic web standards.” This sentence should be “A community of practice and supporting tools have been developed around RDF and related semantic web standards.” This sentence would be good to provide examples of these tools and highlight which aspects of RDF are being worked on. Other sentences need to be divided into shorter sentences. For example, the sentence “Previous studies in the field of knowledge graphs focused on static data, however, methods to deal with and capture the variation and development of data over time, is of high importance and little is known about presenting temporal data in knowledge graphs” is way too long and it needs to be divided into at least two smaller sentences if not three sentences. Some concepts are not used correctly, like “improve the performance of KG models,” a knowledge graph doesn’t have performance. Therefore, are the models in question the creation process or the querying? They need to be explained better. The last penultimate paragraph of the introduction needs to be reordered. The paragraph tries to summarize the aim of the paper, but it jumps back in forth between the purpose of the paper and the benefits of using temporal data in a health case setting. Finally, the last paragraph doesn’t close the introduction well. I would recommend restructuring the penultimate paragraph for the closing of the paragraph. The second main negative issue is that even though the methodology behind the study is well-defined, the results of the study aren’t actually presented. Therefore, the actual impact of the study can only be assumed but not confirmed.

 This paper presents a well-defined study to find existing works illustrating the benefits and challenges of modeling temporal data. Unfortunately, the paper needs rewriting in some parts, as well as fixing some typos. 

**Section Comments**

**Section 1: Introduction**

- Positive: This section does a good job of introducing the reader to the purpose of this work, as well as the needed background knowledge to understand this work.
- Negative: Some sentences in the introduction need to be rewritten. Some sentences could be more understandable. For example, “A community of practice and supporting tools have developed around the RDF and related semantic web standards.” This sentence should be “A community of practice and supporting tools have been developed around RDF and related semantic web standards.” This sentence would be good to provide examples of these tools and highlight which aspects of RDF are being worked on. Other sentences need to be divided into shorter sentences. For example, the sentence “Previous studies in the field of knowledge graphs focused on static data, however, methods to deal with and capture the variation and development of data over time, is of high importance and little is known about presenting temporal data in knowledge graphs” is way too long and it needs to be divided into at least two smaller sentences if not three sentences. Some concepts are not used correctly, like “improve the performance of KG models,” a knowledge graph doesn’t have performance. Therefore, are the models in question the creation process or the querying? They need to be explained better. In some cases where items are being listed do not have an “and” in the last item. For example, “natural language processing, question answering machines, recommendation system” doesn’t have an “and” between question answering machines and recommendation system. This can also be seen in “OpenEHR, HL7.” The acronym for knowledge graph is introduced twice (one right after the other) and is only used two more times afterward and never again in the rest of the paper. The last penultimate paragraph of the introduction needs to be reordered. The paragraph tries to summarize the aim of the paper, but it jumps back in forth between the purpose of the paper and the benefits of using temporal data in a health case setting. Finally, the last paragraph doesn’t close the introduction well. I would recommend restructuring the penultimate paragraph for the closing of the paragraph.

**Section 2: Protocol**

- Positive: This section defines all the characteristics of the study that will conducted, including the databases used to search for the relevant papers, queries used for each particular database, and the criteria in which the papers are considered for the study.
- Negative: This section presents a couple of sentences I recommend changing to make them cleaner. “The bibliographies of the included full-text articles will be searched for relevant articles” would be better written as “The bibliographies of the included full-text articles will be searched for additional relevant articles.”  In the sentence “The search strategy was developed using the PEO (Population (Context), Exposure, and Outcome) framework as follows,” it’s strange to have parentheses within parentheses. Finally, the sentence “The data to be extracted in the table” should be “The data to be extracted into the table.”

**Section 3: Conclusion**

- Positive: This section closes the paper well. It is a good summary of what is presented in the paper and its possible impact.
- Negative: The main issue with this section is that the paper doesn’t have any results to support the impact of the study. Therefore, any conclusion drawn from the paper can only be considered a prediction. Additionally, there is a small typo in “organisations”; it should be “organizations.”

Is the study design appropriate for the research question?

Yes

Is the rationale for, and objectives of, the study clearly described?

Yes

Are sufficient details of the methods provided to allow replication by others?

Yes

Are the datasets clearly presented in a useable and accessible format?

Not applicable

Reviewer Expertise:

Knowledge graph creation, Data managment

I confirm that I have read this submission and believe that I have an appropriate level of expertise to confirm that it is of an acceptable scientific standard, however I have significant reservations, as outlined above.

# Reviewer report: Reviewer response for version 2

**DOI:** 10.21956/hrbopenres.14859.r32619

**Published:** 2022-09-15

**Authors:** Bridget Kane

**Affiliations:** 1 Karlstad University, Karlstad, Sweden

I have reviewed the changes. I am satisfied with the amendments made by the authors and I formally approve this publication.

Is the study design appropriate for the research question?

Yes

Is the rationale for, and objectives of, the study clearly described?

Partly

Are sufficient details of the methods provided to allow replication by others?

Partly

Are the datasets clearly presented in a useable and accessible format?

Not applicable

Reviewer Expertise:

Health Information Systems, eHealth, human-computer interaction (HCI)  in healthcare and computer-supported collaborative work (CSCW) in healthcare.

I confirm that I have read this submission and believe that I have an appropriate level of expertise to confirm that it is of an acceptable scientific standard.

# Reviewer report: Reviewer response for version 1

**DOI:** 10.21956/hrbopenres.14603.r30875

**Published:** 2021-12-14

**Authors:** Paul Stacey

**Affiliations:** 1 School of Informatics and Engineering, Technological University Dublin, Dublin, Ireland

This article proposes an interesting protocol for a systematic review to investigate current approaches to the modelling of temporal data in knowledge graphs. The protocol is based on pre-existing guidelines, but is unique in it's application to establishing a review of existing approaches in modelling temporal data in knowledge graphs and some of the underlying methods employed within the protocol.

 The purpose of the review is to ultimately investigate the use of knowledge graphs in representing, storing and managing complex healthcare data. However, this will be the focus of future work. This article describes the approach to be taken to determine the current body of work in using knowledge graphs and specifically RDF to handle temporal models or the application of the latter to the former. From that perspective the article is of interest to the health informatics research community. 

 The article is well written and structured well, allowing the reader to easily navigate the material and follow the argument and the protocol proposed. However, there are a few areas within the article that should be reviewed to aide clarity for the reader. These are outlined below:

- The introduction makes a big jump from stating the need for effective methods for healthcare data storage and representation and in particular dealing with temporal data without mentioning some key approaches already employed. The argument for the "need" isn't well established in the text at this point. 
- Knowledge Graphs are proposed as a solution somewhat abruptly. It would be helpful for the reader to include some additional background here on the challenges that current approaches suffer when representing temporal data and why KGs could be the solution.
- The term healthcare data is very broad, and there are examples of using knowledge graphs within the literature for some specific use cases and sub sets of healthcare data. But is the focus on Patient data/information? Or perhaps it is all healthcare data and a big data approach? I suspect it is all data given the reference material to big data source material, however this is not overly clear in the text.
- Some additional rationale should be given for the selection of terms for the search strategy. 
- The research question states: "What are the existing approaches in modelling temporal data in knowledge graphs?" - but the study is only focused on articles mentioning RDF. However, RDF is not the only way to represent knowledge graphs as is acknowledged later in the text. Therefore the research question should be narrowed or the study design broadened to all knowledge graphs or at least a rationale for only focusing on RDF should be provided for the reader.
- The statement "However, little is known about the use or suitability for use with healthcare data." is a very broad statement and the literature would show that there is a reasonable, if not limited body of knowledge available in the literature investigating KGs applicability to healthcare data . But, again, perhaps it is the particular type of healthcare data that the authors are referring to, this is unclear and should be reviewed. 
- The authors make the argument that temporal data is important to be represented, but don't link this to healthcare data approaches already employed within the health informatics community to represent temporal data, for example: Data Types Information Model (openehr.org). What are the limitations of these approaches and why are knowledge graphs important etc? Or perhaps the author seeks to apply these approaches to knowledge graphs and the reification approaches used such as RDF; this is unclear from the reader's perspective. 
- The statement "The results of the systematic review will inform data engineers when modelling temporal data". This statement is somewhat reductive and should be broadened to something like "informaticians and the data science community".
- Would the authors consider using Scopus instead of ScienceDirect as one of the information sources? Scopus would give a broader coverage from additional publishers beyond that of ScienceDirect, while also covering the metadata from ScienceDirect. 
- Reference 28 and 29 appear to link to the same Figure?
- Some minor grammatical errors and typos exist that should be reviewed, for example: In the Abstract space required between "time”.Data". 

Is the study design appropriate for the research question?

Partly

Is the rationale for, and objectives of, the study clearly described?

Partly

Are sufficient details of the methods provided to allow replication by others?

Partly

Are the datasets clearly presented in a useable and accessible format?

Not applicable

Reviewer Expertise:

Information modelling, semantic systems, knowledge based systems, internet of things.

I confirm that I have read this submission and believe that I have an appropriate level of expertise to confirm that it is of an acceptable scientific standard, however I have significant reservations, as outlined above.

# Response

**Published:** 2022-07-28

**Authors:** Sepideh Hooshafza

**Affiliations:** Trinity College Dublin, Ireland

**This article proposes an interesting protocol for a systematic review to investigate current approaches to the modelling of temporal data in knowledge graphs. The protocol is based on pre-existing guidelines, but is unique in it's application to establishing a review of existing approaches in modelling temporal data in knowledge graphs and some of the underlying methods employed within the protocol.**

**The purpose of the review is to ultimately investigate the use of knowledge graphs in representing, storing and managing complex healthcare data. However, this will be the focus of future work. This article describes the approach to be taken to determine the current body of work in using knowledge graphs and specifically RDF to handle temporal models or the application of the latter to the former. From that perspective the article is of interest to the health informatics research community.**

**The article is well written and structured well, allowing the reader to easily navigate the material and follow the argument and the protocol proposed. However, there are a few areas within the article that should be reviewed to aide clarity for the reader. These are outlined below:**

**1. The introduction makes a big jump from stating the need for effective methods for healthcare data storage and representation and in particular dealing with temporal data without mentioning some key approaches already employed. The argument for the "need" isn't well established in the text at this point.**

 Thank you for your comment. We have now included additional text to clarify the need for temporal data modelling specifically in healthcare with a brief introduction of temporal data in healthcare, as below:

*“Most healthcare data are temporal in nature* *.* *^(10)^* *Most of the clinical data such as patients’ symptoms, laboratory test results, data from health monitoring devices, and various clinical measurements, are associated with a time in which the data is valid (valid time). Furthermore, capturing the time that the data are inserted into the database (transaction time) is required for legal purposes, treatment purposes or for justifying physicians’ decisions.* *^(10)^* *As a simple example, suppose a patient has been hospitalised on February 18th at 8:00 am. This is the valid time for the hospitalisation time. This piece of information is inserted into hospital database on Feb 18 at 12:00 pm. This time is called the transaction time* *.* *^(10)^* *Capturing both transaction time and valid time is important to ensure that the decision making process is accurate and valuable and is beneficial in designing decision support systems.* *^(11)^*

*Improved management of temporal healthcare data would benefit healthcare practitioners in information retrieval, healthcare decision making and support patient care.* *^(12)^* *Temporal data can assist in exploring temporal patterns in diseases and identifying disease progression. It would help in assessing patients’ clinical history, finding possible causes of clinical events over time, and predicting future trends and events based on past and current clinical data.* *^(2)^* *Hence, the representation and query of temporal data has become a priority research area* *and efficient solutions are needed to model and store temporal data in healthcare settings.* *^(13,14)^* *“*

 And

*“Temporal data models are a way to ensure time* *consistency in data models and assist analysing the history of data and predicting the future trends in data.* *The management of rapidly changing patient data and types of data sets is an ongoing challenge. This study focuses on the approaches and challenges in KG modelling to support this management.* *^(3, 4)^* *”*

**2. Knowledge Graphs are proposed as a solution somewhat abruptly. It would be helpful for the reader to include some additional background here on the challenges that current approaches suffer when representing temporal data and why KGs could be the solution.**

 Thank you for your comments. We have included the following text to address this comment:

*“Most of the solutions implemented so far for storing, and representing valid time and transaction time are based on relational databases. The developers of clinical database systems have some difficulty managing time values in relational databases for highly connected data. Firstly, modelling relationships and semantics between data items is not easy to implement in relational databases.* *^(15)^* *Secondly, for the large volume of data coming from heterogeneous sources, it is difficult to query a large number of joining tables.* *^(16)^* *As such, knowledge graphs (KGs) are potential solution to these problems.”*

**3. The term healthcare data is very broad, and there are examples of using knowledge graphs within the literature for some specific use cases and sub sets of healthcare data. But is the focus on Patient data/information? Or perhaps it is all healthcare data and a big data approach? I suspect it is all data given the reference material to big data source material, however this is not overly clear in the text.**

 We apologise that this was not clear. We mean all healthcare data including patient data, data from health monitoring devices, data collected within services. We have included some examples of healthcare data in the introduction to clarify this further:

*“Most healthcare data are temporal in nature* *.* *^(10)^* *Most of the clinical data such as patients’ symptoms, laboratory test results, data from health monitoring devices, and various clinical measurements, are associated with a time in which the data is valid (valid time). Furthermore, capturing the time that the data are inserted into the database (transaction time) is required for legal purposes, treatment purposes or for justifying physicians’ decisions.* *^(10)^* *As a simple example, suppose a patient has been hospitalised on February 18th at 8:00 am. This is the valid time for the hospitalisation time. This piece of information is inserted into hospital database on Feb 18 at 12:00 pm. This time is called the transaction time* *.* *^(10)^* *Capturing both transaction time and valid time is important to ensure that the decision making process is accurate and valuable and is beneficial in designing decision support systems.* *^(11)^*

**4. Some additional rationale should be given for the selection of terms for the search strategy.**

 We have included additional text in the search strategy section as follows:

*“The search strategy was developed using the PEO (Population (Context), Exposure, and Outcome) framework as follows, Population (Context): knowledge graph, Exposure: Time, and Outcome: Applied model. The search concepts and keywords based on PEO framework are set out in* *Table 1* *.*

*Table* *1* *-* *Search terms for a systematic review on modelling temporal data in knowledge graphs*

PEO framework

Concepts

Search terms

Population (Context)

 knowledge graph

 “Knowledge graph” OR RDF OR “resource description framework”

Exposure

 Time

 Temporal OR dynamic OR evolution OR time

Outcome

 Applied model

 *present OR annotate OR model OR schema OR standard OR framework OR structure OR application OR applied

**5. The research question states: "What are the existing approaches in modelling temporal data in knowledge graphs?" - but the study is only focused on articles mentioning RDF. However, RDF is not the only way to represent knowledge graphs as is acknowledged later in the text. Therefore the research question should be narrowed or the study design broadened to all knowledge graphs or at least a rationale for only focusing on RDF should be provided for the reader.**

 Thank you for your comment, we have updated the research question as follows:

*“What are the existing approaches in modelling temporal data in RDF based knowledge graphs?”*

 There a number of reasons why the focus was on RDFs. We have also included text that highlights the rationale for using RDF graphs in the introduction to justify the focus of the review.

*“For a number of reasons, the focus of this review is on RDF based knowledge graphs. RDF is a standard language for data representation and interchange on the Web.* *^(21)^* *RDF graphs are popular in practice and follow the World Wide Web Consortium (W3C) standards.* *^(22)^* *A community of practice and supporting tools have developed around the RDF and related semantic web standards. Many standards based public data models (called ontologies) are available to support and guide RDF KG enhancement and development.”*

**6. The statement "However, little is known about the use or suitability for use with healthcare data." is a very broad statement and the literature would show that there is a reasonable, if not limited body of knowledge available in the literature investigating KGs applicability to healthcare data. But, again, perhaps it is the particular type of healthcare data that the authors are referring to, this is unclear and should be reviewed.**

 We agree with the reviewer and apologise that we were not tighter with the language. What was meant was limited data in relation to temporal healthcare data specifically. We have removed this statement and replaced it with:

 “ *Little is known about presenting temporal data in knowledge graphs*.”

**7. The authors make the argument that temporal data is important to be represented, but don't link this to healthcare data approaches already employed within the health informatics community to represent temporal data, for example: Data Types Information Model (openehr.org). What are the limitations of these approaches and why are knowledge graphs important etc? Or perhaps the author seeks to apply these approaches to knowledge graphs and the reification approaches used such as RDF; this is unclear from the reader's perspective.**

 Thank you for your comment, we have included additional text in the introduction to address this. The additional text is as follows:

*“There are different international health information modelling standards including OpenEHR, HL7 which are used for modelling information at high level and for semantic interoperability purposes. In this review our focus is on the modelling in the data level, where we can model data to store them in the database and query them. International health information modelling standards can be used to get a high level overview of the concept with its included information items and can be used for designing different concepts in knowledge graphs but they are not replaceable.”*

**8. The statement "The results of the systematic review will inform data engineers when modelling temporal data". This statement is somewhat reductive and should be broadened to something like "informaticians and the data science community".**

 Thank you for your comment, we agree and we have now included the informatics area in the conclusion section as well, as follows:

*“The findings of the systematic review will be of interest to organisations working in the fields of data science, data engineering, informatics and other similar areas.”*

**9. Would the authors consider using Scopus instead of ScienceDirect as one of the information sources? Scopus would give a broader coverage from additional publishers beyond that of ScienceDirect, while also covering the metadata from ScienceDirect.**

 Thank you very much for your suggestion. We have reviewed the content of both databases and we agree with changing ScienceDirect to Scopus since it is more comprehensive. We have updated the protocol as follow:

*"Searches will be carried out on the following databases: ACM Digital Library ^(27)^, IEEE Xplore Digital Library ^(28)^, and Scopus ^(29)^."*

**10. Reference 28 and 29 appear to link to the same Figure?**

 Thank you for highlighting this, we apologise for this error. We have corrected this error.

**11. Some minor grammatical errors and typos exist that should be reviewed, for example: In the Abstract space required between "time”. Data".**

 Thank you for highlighting this. We have corrected this error. We have also reviewed the paper in full for grammatical errors and typos.

# Reviewer report: Reviewer response for version 1

**DOI:** 10.21956/hrbopenres.14603.r30297

**Published:** 2021-11-08

**Authors:** Bridget Kane

**Affiliations:** 1 Karlstad University, Karlstad, Sweden

This is an interesting protocol to undertake a systematic review on modelling temporal data in knowledge graphs.

 Generally, the study is well described. However, it would be better if more detail could be given about the Resource Development Framework (RDF), its importance and the reasons why it is a critical inclusion criterion. Please also expand on why studies based on other frameworks will be excluded.

 Other points that should be addressed:

- Information sources: Is there a reason why PubMed is not included? 
- Search strategy: Isn't the idea of publishing a protocol is that the exact protocol is provided and can be replicated? Yet, the search strategy given here is called 'an example'.
- The search strategy should be definitive and clearly articulated.
- Rather than assume that data from health monitoring devices are included, it would be good to be more clear, i.e. explicitly state that knowledge graphs of such data are included. 

Is the study design appropriate for the research question?

Yes

Is the rationale for, and objectives of, the study clearly described?

Partly

Are sufficient details of the methods provided to allow replication by others?

Partly

Are the datasets clearly presented in a useable and accessible format?

Not applicable

Reviewer Expertise:

Health Information Systems, eHealth, human-computer interaction (HCI)  in healthcare and computer-supported collaborative work (CSCW) in healthcare.

I confirm that I have read this submission and believe that I have an appropriate level of expertise to confirm that it is of an acceptable scientific standard, however I have significant reservations, as outlined above.

# Response

**Published:** 2022-07-28

**Authors:** Sepideh Hooshafza

**Affiliations:** Trinity College Dublin, Ireland

**This is an interesting protocol to undertake a systematic review on modelling temporal data in knowledge graphs.**

**1. Generally, the study is well described. However, it would be better if more detail could be given about the Resource Development Framework (RDF), its importance and the reasons why it is a critical inclusion criterion. Please also expand on why studies based on other frameworks will be excluded.**

 Thank you for your comment. There are a number of reasons why the focus was on RDFs. We have included the following text in the introduction that highlights the rationale for using RDF graphs to justify the focus of the paper.

*“For a number of reasons, the focus of this review is on RDF based knowledge graphs. RDF is a standard language for data representation and interchange on the Web.* *^(21)^* *RDF graphs are popular in practice and follow the World Wide Web Consortium (W3C) standards.* *^(22)^* *A community of practice and supporting tools have developed around the RDF and related semantic web standards. Many standards based public data models (called ontologies) are available to support and guide RDF KG enhancement and development.”*

**Other points that should be addressed:**

**2. Information sources: Is there a reason why PubMed is not included?**

 PubMed is a repository focusing on life sciences and biomedical topics. We decided to include computer science databases and explore the area in general and not specifically in healthcare settings. The aim was to give a general view of the approaches, applications and challenges of temporal data modelling in knowledge graphs. We have as such not included PubMed despite the reviewer’s observation.

**3. Search strategy: Isn't the idea of publishing a protocol is that the exact protocol is provided and can be replicated? Yet, the search strategy given here is called 'an example'.**

 Thank you for your comment. We were following the PRISMA guidelines on providing an example of a search for one database. However, the PRISMA guidelines have been updated and now suggest including all searches in the protocol. As such we have now included the exact search strategy in the protocol and we have removed the word “example” from the text.

*“The search query to be used for ACM digital library, and Scopus, is:*

((Temporal OR dynamic OR evolution OR time) AND (“Knowledge graph” OR rdf OR “resource description framework”) AND (*present OR annotate OR model OR schema OR standard OR framework OR structure OR application OR applied))

*Since IEEE Xplore Digital Library does not accept using parenthesis in the advanced search, the search query to be used is:*

*“Knowledge graph” OR rdf OR “resource description framework”*

*AND*

*Temporal OR dynamic OR evolution OR time*

*AND*

*present OR annotate OR model OR schema OR standard OR framework OR structure OR application OR applied “

**4. The search strategy should be definitive and clearly articulated.**

 As above, the exact search strategy has now been written in the protocol.

*“The search query to be used for ACM digital library, and Scopus, is:*

((Temporal OR dynamic OR evolution OR time) AND (“Knowledge graph” OR rdf OR “resource description framework”) AND (*present OR annotate OR model OR schema OR standard OR framework OR structure OR application OR applied)) “

*Since IEEE Xplore Digital Library does not accept using parenthesis in the advanced search, the search query to be used is:*

*“Knowledge graph” OR rdf OR “resource description framework”*

*AND*

*Temporal OR dynamic OR evolution OR time*

*AND*

*present OR annotate OR model OR schema OR standard OR framework OR structure OR application OR applied “

**5. Rather than assume that data from health monitoring devices are included, it would be good to be clearer, i.e. explicitly state that knowledge graphs of such data are included.**

 We did not want to specify health monitoring devices because that level of specificity may have skewed the study where we are attempting to cover all health data. However, we acknowledge take your point and have included health care monitoring devices in the list of examples so as to be more explicit, as follows:

*“Most healthcare data are temporal in nature.* *^(10)^* *Most of the clinical data like patients’ symptoms, laboratory test results, data from health monitoring devices, and various clinical measurements, are associated with a time in which the data is valid (valid time).”*
